# RETRACTION: Effect of Bone Grafting on Postoperative Wound Infection and Marginal Necrosis in Patients With Calcane Fractures

**DOI:** 10.1111/iwj.70395

**Published:** 2025-03-21

## Abstract

**RETRACTION:** YeL. , SuY. , FanY. , and WuM. , “Effect of Bone Grafting on Postoperative Wound Infection and Marginal Necrosis in Patients With Calcane Fractures,” International Wound Journal 21, no. 2 (2024): e14660, 10.1111/iwj.14660.PMC1083091942052968

The above article, published online on 31 January 2024, in Wiley Online Library (http://onlinelibrary.wiley.com/), has been retracted by agreement between the journal Editor in Chief, Professor Keith Harding; and John Wiley & Sons Ltd. Following an investigation by the publisher, all parties have concluded that this article was accepted solely on the basis of a compromised peer review process. The editors have therefore decided to retract the article. The authors did not respond to our notice regarding the retraction.

**RETRACTION:** L. Ye , Y. Su , Y. Fan , and M. Wu , “Effect of Bone Grafting on Postoperative Wound Infection and Marginal Necrosis in Patients With Calcane Fractures,” International Wound Journal 21, no. 2 (2024): e14660, 10.1111/iwj.14660.PMC1083091942052968

The above article, published online on 31 January 2024, in Wiley Online Library (http://onlinelibrary.wiley.com/), has been retracted by agreement between the journal Editor in Chief, Professor Keith Harding; and John Wiley & Sons Ltd. Following an investigation by the publisher, all parties have concluded that this article was accepted solely on the basis of a compromised peer review process. The editors have therefore decided to retract the article. The authors did not respond to our notice regarding the retraction.

